# The atherogenic index of plasma is associated with an increased risk of diabetes in non-obese adults: a cohort study

**DOI:** 10.3389/fendo.2024.1477419

**Published:** 2025-01-20

**Authors:** Jun Cao, Zhaohai Su, Jiangyong Yang, Bilong Zhang, Rengui Jiang, Weiling Lu, Zhenhua Huang, Zheng Xie

**Affiliations:** ^1^ Department of Cardiology, Guangdong Provincial People’s Hospital Ganzhou Hospital, Ganzhou Municipal Hospital (Gannan Medical University Affiliated Municipal Hospital), Ganzhou, China; ^2^ Department of Emergency Medicine, The First Affiliated Hospital of Shenzhen University, Shenzhen Second People’s Hospital, Shenzhen, China; ^3^ Department of Emergency Medicine, Pengpai Memorial Hospital, Shanwei, China; ^4^ Department of General Practice, Guangdong Provincial People’s Hospital Ganzhou Hospital, Ganzhou Municipal Hospital (Gannan Medical University Affiliated Municipal Hospital), Ganzhou, China

**Keywords:** atherogenic index of plasma, diabetes, non-obese, Chinese adults, non-linear

## Abstract

**Objective:**

This study aims to investigate the relationship between the atherogenic index of plasma (AIP) and diabetes risk in Chinese non-obese adults. This is important because the incidence of diabetes is significant in non-obese populations, and evidence regarding this association is limited.

**Methods:**

We conducted a retrospective cohort study with 82,977 Chinese non-obese adults. We used Cox proportional hazards regression to assess the relationship between baseline AIP levels and diabetes incidence. We also employed cubic spline functions and smooth curve fitting to investigate potential nonlinear relationships. Sensitivity and subgroup analyses were conducted to validate our findings.

**Results:**

The median follow-up duration for these participants was 3.10 years, during which 1,041 subjects (1.25%) were diagnosed with diabetes. Adjusted analyses demonstrated a strong positive association between AIP and the risk of diabetes onset (HR 2.07; 95% CI: 1.63-2.63; p < 0.001). The risk of diabetes increased with higher AIP quartiles, especially between the highest (Q4) and lowest (Q1) quartiles (adjusted HR 1.55; 95% CI: 1.27-1.89). We also identified a nonlinear relationship between AIP and diabetes risk. Sensitivity and subgroup analyses confirmed these findings. Furthermore, E-value analysis indicated that the results were robust against unmeasured confounding variables.

**Conclusion:**

Our findings highlight a positive, nonlinear association between AIP and diabetes risk in Chinese non-obese adults. Lowering triglycerides (TG) or increasing high-density lipoprotein cholesterol (HDL-C) levels may help reduce this risk.

## Introduction

Diabetes mellitus (DM) is a significant global public health issue, characterized by chronic metabolic dysregulation due to insulin resistance and inadequate insulin secretion (1, 2). Recent statistics from 2019 show that around 463 million people globally suffer from type 2 diabetes, projected to increase to approximately 700 million by 2045 (3). Notably, China has the highest global diabetes prevalence, with an estimated 140 million cases in 2021 (4). Diabetes has multifaceted implications, including vascular damage that accelerates atherosclerosis and increases cardiovascular disease risk, and renal damage that may lead to kidney failure, requiring dialysis or transplantation in severe cases. Additionally, diabetic retinopathy, caused by damage to the retinal microvasculature, can result in visual impairment or blindness, significantly burdening affected individuals (5–8). Early identification of risk factors is crucial for controlling the rapid increase in diabetes (9).

A study indicated that elevated HSI is closely associated with a higher risk of T2DM in the Chinese population (10). Recent studies have indicated that abnormal lipid metabolism includes hypercholesterolemia, elevated low-density lipoprotein cholesterol (LDL-C), hypertriglyceridemia, and reduced high-density lipoprotein cholesterol (HDL-C), all of which are associated with an increased risk of cardiovascular diseases (11, 12). All forms of abnormal lipid metabolism, whether occurring alone or in combination, are linked to an increased risk of diabetes (13). Accordingly, we know that the atherogenic index of plasma (AIP), which reflects atherogenic dyslipidemia, is an important marker for assessing the risk of atherosclerosis, insulin resistance, cardiovascular diseases, and metabolic syndrome (14–16 Some studies have found a significant relationship between AIP and diabetes risk (17, 18). However, these studies have primarily focused on the general population as well as individuals with overweight and obesity, with relatively few studies on young non-obese adults. Recent findings indicate that the prevalence of diabetes among non-obese individuals can be as high as 2.8% (19). An epidemiological study showed that Asian individuals with ectopic fat obesity defined by fatty liver have a significantly higher risk of type 2 diabetes even at lower body mass index (BMI) levels compared to other obesity types, such as obesity (BMI ≥ 25 kg/m²) (20). This suggests that diabetes in the non-obese population, especially among Asians, should not be overlooked.

Considering this, we explore the relationship between AIP and diabetes risk among non-obese adults based on a large cohort study of the Chinese population. This study aims to better understand the diabetes risk factors in this population and develop effective preventive strategies.

## Methods

### Study design and population

This study employed a retrospective cohort design, utilizing data from a Chinese computer database by researchers Chen et al. (21). The original data for this study were sourced from the DATADRYAD database (www.datadryad.org). The data concerning Chinese individuals came from a published article entitled “Association of body mass index and age with incident diabetes in Chinese adults: a population-based cohort study” referred to as the Dryad dataset (https://doi.org/10.5061/dryad.ft8750v) (21). This research respected the principles outlined in the Declaration of Helsinki, with all procedures aligning with the relevant protocols and rules as specified in the declaration segment. As a result of its retrospective design, ethical consent or informed approval was not necessary from the institutional review board for the analysis of this secondary dataset. Dryad’s terms of service permit the secondary analysis of data by other researchers without infringing upon the authors’ rights.

**Figure 1:**
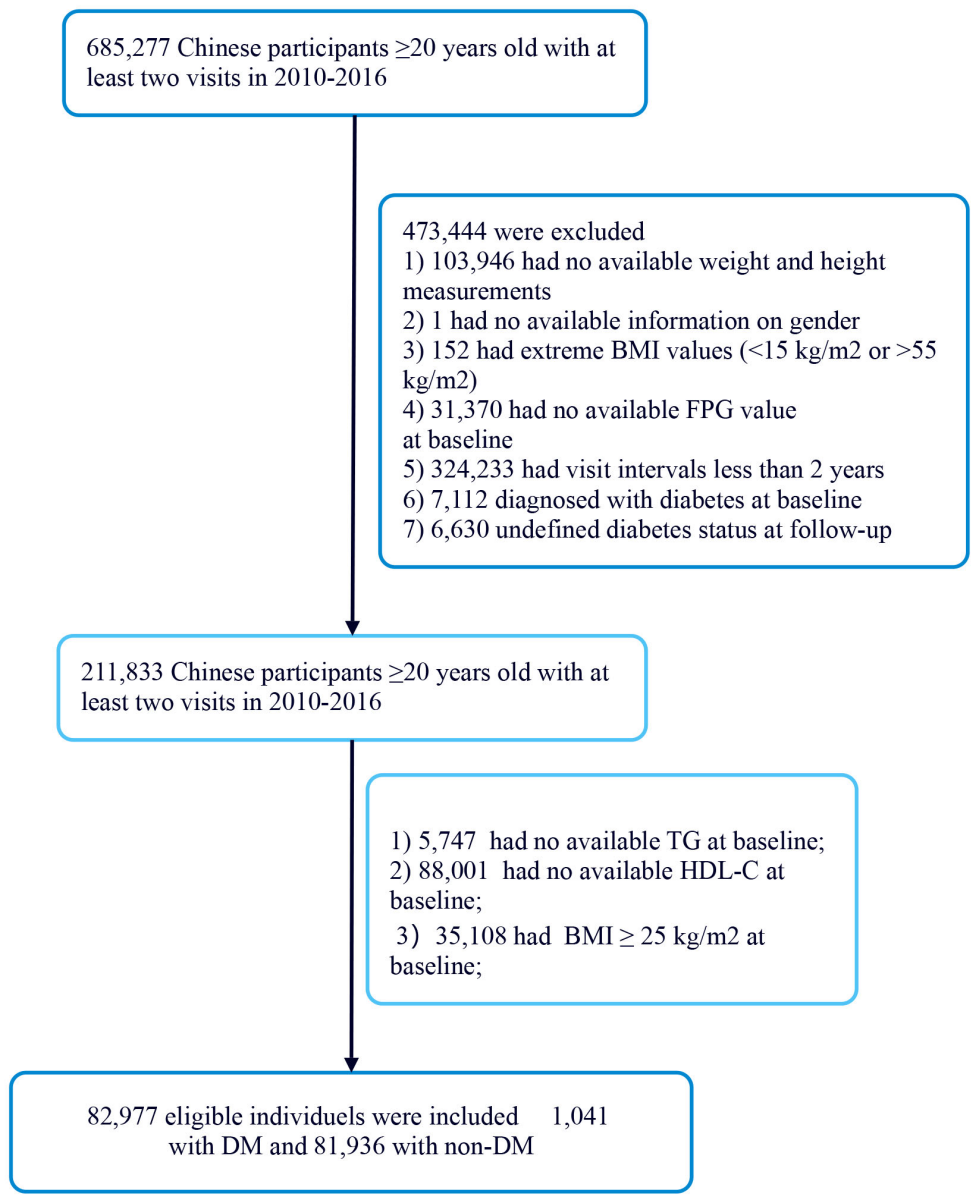
Flowchart of study participants.

**Figure 1** illustrates the initial inclusion of 685,277 participants in the Chinese cohort, with 473,744 excluded from the primary study, leaving 211,833 for analysis. The exclusion criteria for the current analysis were as follows: (i) participants lacking baseline TG data were excluded (n=5,748); (ii) participants lacking baseline HDL-C data were excluded (n=88,001); (iii) participants had BMI≥25kg/m2 (Non-obesity is defined as BMI <25 kg/m^2)^ (20) were excluded (n=35,108); The final participant count was 82,977 comprising 1041 with DM and 81,936 with non-DM.

### Measurement of exposure and outcome measures

The exposure variable in this study is the initial AIP, which is calculated based on the participants’ TG and HDL-C levels using the formula: AIP = log (TG/HDL-c) (22, 23). The criteria for incident DM included self-report and a FPG level ≥7.0 mmol/l measured at the last follow-up evaluation. The earliest result was used as the basis for diagnosis. Additionally, the follow-up period spanned five years, with a minimum follow-up duration of 2 years (21). The primary outcome was determined during the follow-up period and was whether participants were diagnosed with diabetes, which was recorded as a binary variable (0 = non-DM, 1 = DM).

### Covariates

In this study, we collected data from both Chinese populations, focusing on shared variables such as demographic characteristics (age and gender), fasting plasma glucose (FBG), BMI, alanine aminotransferase (ALT), systolic blood pressures (SBP), TG, total cholesterol (TC), diastolic blood pressures (DBP), HDL-c, blood urea nitrogen (BUN), serum creatinine (Scr), family history of diabetes, as well as follow-up duration. We also collected smoking status, categorized as current smoker, former smoker, never smoker, and unknown. Additionally, we collected alcohol consumption status, categorized as current drinker, former drinker, never drinker, and unknown. Fasting venous blood samples were collected after a minimum 10-hour fast at each visit. Blood pressure was measured using a standard mercury sphygmomanometer. BMI was calculated as weight in kilograms divided by the square of height in meters. Covariates were selected based on clinical experience and published literature, leading us to include the following variables: continuous variables such as DBP, FBG, BMI, HDL-C, SBP, TG, TC, and ALT; and categorical variables such as gender, smoking status, drinking status and family history of diabetes.

### Missing data processing

In this study, the number of participants with missing data for DBP, SBP, TC, LDL, ALT, BUN, and Scr were 9 (0.00%), 9 (0.00%), 1 (0.00%), 0 (0.00%), 301 (0.36%), 2170 (2.62%), and 979 (1.17%), respectively. The study used multiple imputation to handle the missing data to reduce the variability caused by missing variables. DBP, SBP, age, ALT, gender, LDL-c, TG, HDL, Scr, BUN, TC, FPG, alcohol consumption status, smoking status, and family history of diabetes were all included in the imputation model (24).

### Statistical analysis

The study included participants who were classified into four groups based on their AIP values. The mean ± standard deviation (for normally distributed data) or medians (with interquartile ranges) (for skewed data) were reported for continuous variables. Categorical data was presented as frequencies and percentages. The analysis involved using the χ2 test for categorical variables, and either one-way analysis of variance (ANOVA) (for normal data) or the Kruskal-Wallis H test (for skewed data) to compare differences between the AIP groups. Survival rates and time-to-event variables were determined through the Kaplan-Meier method, and the log-rank test was employed to compare diabetes-free survival among the AIP groups.

We used univariate and multivariate Cox proportional hazards regression models to investigate the association between AIP and diabetes risk. This included a crude model without adjusted covariates, a model adjusted for the minimum covariates (Model I: adjusted for gender and age), and a fully adjusted model (Model II: adjusted for gender, age, SBP, DBP, BMI, family history of diabetes, drinking status, smoking status, LDL-C, ALT, Scr, BUN, and FPG). We recorded the effect size (HR) and its 95% confidence interval (CI). We adjusted for confounding factors based on clinical experience, literature reports, and the results of univariate analysis. Additionally, to reduce the impact of variables on model stability, we performed collinearity screening and found that the VIF (variance inflation factor) for TC was 8.1, which is greater than 5, indicating a potential collinearity issue (25). Therefore, TC was excluded from the final multivariate Cox proportional hazards regression equation (**Supplementary Table S1**).

Furthermore, using cubic spline functions and smooth curve fitting methods within the Cox proportional hazards regression model, we considered the nonlinear association between AIP and diabetes risk. We also employed a piecewise Cox proportional hazards regression model to elucidate the nonlinear association between AIP and diabetes risk. Finally, we conducted a likelihood ratio test to select the best model to explain their association in non-obese populations. We performed subgroup analyses using stratified Cox proportional hazards regression models across different groups (age, gender, BMI, SBP, DBP, family history of diabetes, smoking status, and drinking status). First, continuous data (such as age) were converted into categorical variables based on clinical cutoffs (age: <65 years, ≥65 years). In addition to the stratifying factors themselves, we adjusted for gender, age, SBP, DBP, BMI, family history of diabetes, drinking status, smoking status, LDL-C, ALT, Scr, BUN, and FPG. Ultimately, in models with and without interaction terms, we used the likelihood ratio test to identify the presence of interaction terms. To examine the reliability of the results, we conducted a series of sensitivity analyses. Continuous covariates were included in the equation and were modeled using generalized additive models (GAM) to confirm the reliability of the findings (26). Additionally, we calculated E-values to assess the potential impact of unmeasured confounding on the association between AIP and diabetes risk (27).

The R software package (http://www.r-project.org, R Foundation) and Empower Stats (X&Y Solutions, Inc., Boston, MA, http://www.empowerstats.com) were utilized for the conducted analyses. Statistical significance was determined with a *P*-value below 0.05.

## Result

### Characteristics of participants

**Table 1** illustrates the demographic and clinical features of the study participants. The mean age was found to be 43.04 ± 12.82 years, with 38,011 individuals (45.81%) classified as male. The median duration of follow-up was 3.10 years, during which time 1041 subjects (1.25%) received a diabetes diagnosis. The AIP ranged from -1.96 to 2.06, resulting in a mean level of -0.42 (**Supplementary Figure S1**). **Table 1** summarizes the baseline characteristics of 82,977 participants, categorized by quartiles of the AIP. The first group included 20,740 participants, the second group had 20,735, the third group consisted of 20,755, and the fourth group comprised 20,747 participants. As AIP increased, the average age of participants significantly rose, with a clear change observed from the first group to the fourth group. Additionally, BMI also increased, reflecting a corresponding trend. SBP and DBP were significantly higher in the fourth group compared to the first group. The FBG levels also showed an upward trend, while TG levels increased substantially. Meanwhile, HDL-C levels exhibited a downward trend across the four groups. The incidence of diabetes increased from 0.70% in Q1 to 2.48% in Q4 (p < 0.001), highlighting a strong association between high AIP and diabetes risk. In addition, we divided the population into a non-DM group and a DM group based on diabetes status. **Supplementary Table S2** presents the baseline characteristics of participants in both groups. Except for alcohol consumption status, all other variables showed significant differences.

**Table 1:** The baseline characteristics of participants.

AIP (quartile)	Q1(-1.96–0.57)	Q2 (-0.57–0.44)	Q3 (-0.44–0.29)	Q4 (-0.29-2.06)	*P*-value
Participants	20740	20735	20755	20747	
Age (years)	40.89 ± 11.79	42.06 ± 12.52	43.62 ± 13.20	45.57 ± 13.24	<0.001
BMI (kg/m^2^)	21.03 ± 2.00	21.39 ± 2.05	21.82 ± 2.01	22.51 ± 1.79	<0.001
SBP (mmHg)	112.94 ± 14.71	115.00 ± 15.43	117.28 ± 15.81	120.53 ± 16.18	<0.001
DBP (mmHg)	70.24 ± 9.73	71.54 ± 9.95	72.94 ± 10.12	75.19 ± 10.47	<0.001
FBG (mmol/L)	4.81 ± 0.55	4.84 ± 0.57	4.89 ± 0.59	4.99 ± 0.61	<0.001
TC (mmol/L)	4.81 ± 0.88	4.69 ± 0.85	4.67 ± 0.88	4.67 ± 0.90	<0.001
TG (mmol/L)	0.60 ± 0.17	0.86 ± 0.22	1.17 ± 0.31	2.08 ± 1.15	<0.001
HDL-c (mmol/L)	1.55 ± 0.31	1.46 ± 0.29	1.39 ± 0.28	1.26 ± 0.28	<0.001
LDL-c (mmol/L)	2.85 ± 0.68	2.74 ± 0.65	2.70 ± 0.66	2.58 ± 0.65	<0.001
ALT (U/L)	14.00 (11.00-18.72)	15.00 (11.30-20.70)	16.50 (12.30-23.40)	20.00 (14.40-28.70)	<0.001
Scr (μmol/L)	64.33 ± 14.02	66.94 ± 15.01	69.48 ± 15.71	72.36 ± 15.60	<0.001
BUN (mmol/L)	4.66 ± 1.19	4.57 ± 1.17	4.59 ± 1.18	4.61 ± 1.15	<0.001
Gender (n, %)					<0.001
Male	5773 (27.84%)	8285 (39.96%)	10568 (50.92%)	13385 (64.52%)	
Female	14967 (72.16%)	12450 (60.04%)	10187 (49.08%)	7362 (35.48%)	
Smoking status (n, %)					
Current smoker	448 (2.16%)	790 (3.81%)	1049 (5.05%)	1577 (7.60%)	
Ever smoker	107 (0.52%)	158 (0.76%)	224 (1.08%)	278 (1.34%)	
Never	4316 (20.81%)	4440 (21.41%)	4627 (22.29%)	4622 (22.28%)	
Unknown	15869 (76.51%)	15347 (74.01%)	14855 (71.57%)	14270 (68.78%)	
Drinking status (n, %)					
Current drinker	53 (0.26%)	93 (0.45%)	123 (0.59%)	201 (0.97%)	
Ever drinker	512 (2.47%)	713 (3.44%)	883 (4.25%)	1177 (5.67%)	
Never	4306 (20.76%)	4582 (22.10%)	4894 (23.58%)	5099 (24.58%)	
Unknown	15869 (76.51%)	15347 (74.01%)	14855 (71.57%)	14270 (68.78%)	
Family history of diabetes, n (%)					0.898
No	20269 (97.73%)	20260 (97.71%)	20297 (97.79%)	20267 (97.69%)	
Yes	471 (2.27%)	475 (2.29%)	458 (2.21%)	480 (2.31%)	
Follow-up (year)	3.14 ± 0.97	3.10 ± 0.95	3.09 ± 0.93	3.08 ± 0.93	<0.001
Incident of diabetes	145 (0.70%)	157 (0.76%)	225 (1.08%)	514 (2.48%)	<0.001

Continuous variables were summarized as mean (SD) or medians (quartile interval); categorical variables were displayed as percentage (%). BMI, body mass index; SBP, systolic blood pressure; DBP, diastolic blood pressure; TG, triglyceride; ALT, alanine aminotransferase; BUN, blood urea nitrogen; Scr, serum creatinine; FBG, fasting plasma glucose; TC, total cholesterol; HDL-c, high-density lipoprotein cholesterol; LDL-c, low-density lipoprotein cholesterol.

### Incidence of diabetes in participants

**Figure 2:**
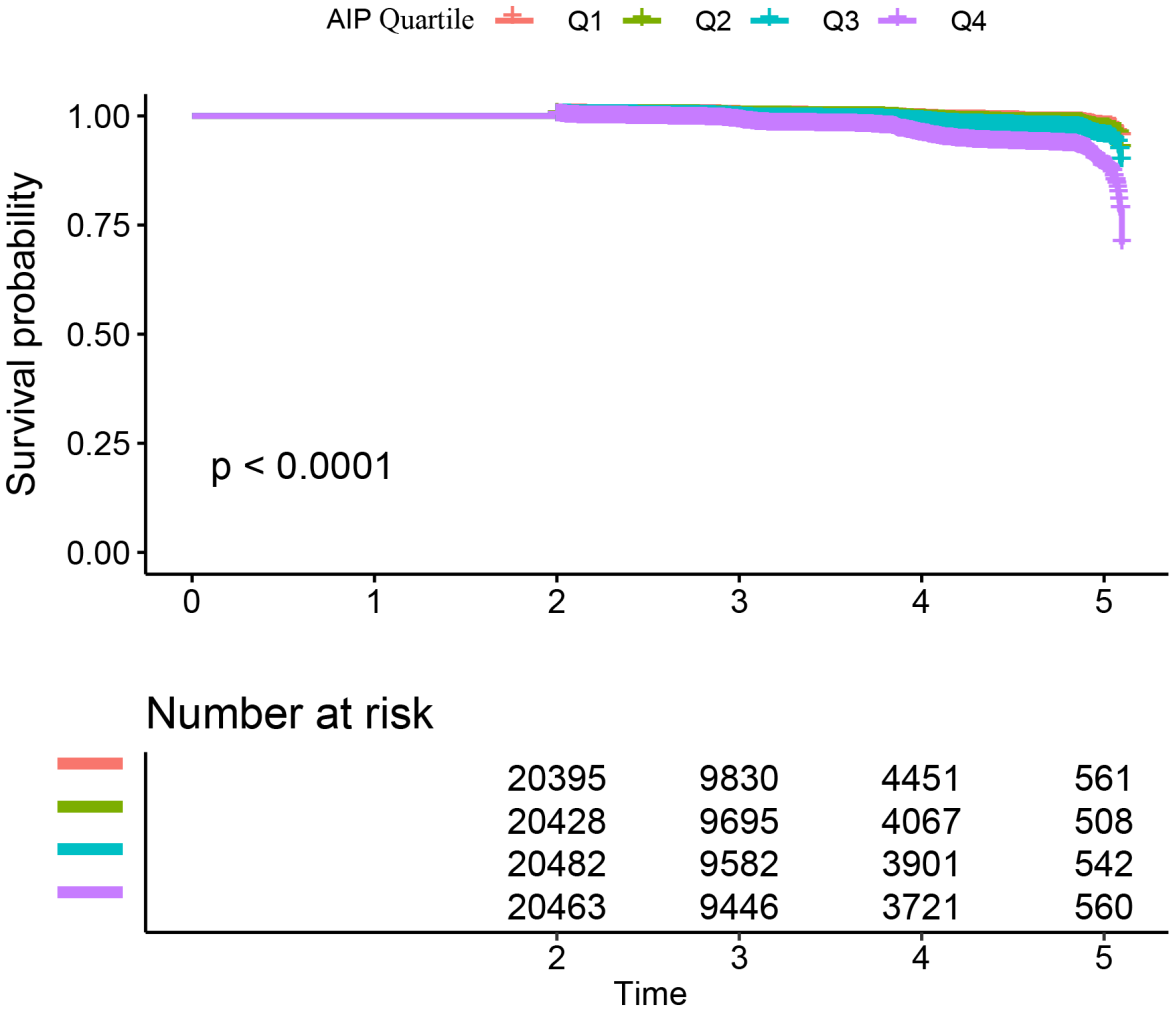
Kaplan–Meier curves for the probability of diabetes. The probability of diabetes increased progressively with rising AIP, meaning that patients with the highest AIP had the higher probability of diabetes in Chinese non-obese adults.

**Table 2** and **Supplementary Figure S2** illustrate the incidence rates of diabetes. Among the study participants, 1,041 individuals (1.25%) were reported to have developed diabetes. The participants were categorized into subgroups according to the quartiles of the AIP. The diabetes incidence rates per 10,000 person-years were recorded as 22.29, 24.52, 34.95, and 80.52 for the respective AIP quartiles. Specifically, the diabetes incidence rates for each quartile were: Q1: 0.70%, Q2: 0.76%, Q3: 1.08%, and Q4: 2.48%. Those participants with the highest AIP (Q4) exhibited an increased risk of diabetes onset in comparison to those with the lowest AIP (Q1) (trend *P*< 0.001). As depicted in **Supplementary Figure S2**, the analysis revealed a significant increase in diabetes prevalence corresponding to the ascending AIP quartiles (*P* < 0.001). **Figure 2** presents the Kaplan-Meier curves, which depict the likelihood of diabetes development based on AIP levels. The transition probabilities were found to differ markedly according to AIP (*P* < 0.001), exhibiting a steady rise in likelihood as the AIP values increased.

**Table 2:** The incidence rate of diabetes (% or Per 10,000 person-year).

AIP (quartile)	Participants (n)	diabetes events (n)	incidence rate(95%CI) (%)	Per 10,000 person-year
Total	82,977	1,041	1.25 (1.18-1.33)	40.32
Q1	20,740	145	0.70 (0.59-0.81)	22.29
Q2	20,735	157	0.76 (0.64-0.88)	24.52
Q3	20,755	225	1.08 (0.94-1.22)	34.95
Q4	20,747	514	2.48 (2.27-2.69)	80.52
P for trend				<0.001

### Factors influencing risk of diabetes analyzed by univariate Cox proportional hazards regression

As shown in **Supplementary Table S3**, the univariate analysis showed that the risk of diabetes was positively associated with DBP, age, BMI, AST, SBP, TG, FPG, ALT, and TC, all with a significance level of *P*<0.05. In addition, AIP was positively associated with risk of diabetes. Conversely, it was negatively associated with HDL-C (all *P*<0.05).

### The association between AIP and diabetes

Three distinct models were created utilizing Cox proportional hazards regression to examine the association between the AIP and the risk of developing diabetes. The first model, which was unadjusted, indicated that for each 1-unit increment in the AIP, there was a 5.93 rise in the probability of advancing to a diabetic condition, reflected by a HR of 6.93(95% CI 5.88-8.16, *P*<0.001). In the second model, which accounted for age and gender only, a 1-unit increase in the AIP corresponded to a 3.96 increase in the probability of diabetes onset, with an HR of 4.96 (95% CI 4.07-6.04, *P*<0.001). The third model, which was fully adjusted, illustrated that a 1-unit increment in the AIP was associated with a 1.07 rise in diabetes likelihood, yielding an HR of 2.07 (95% CI 1.47-2.48, *P*<0.001). The distribution of the confidence intervals underscores the strength of the association between AIP levels and diabetes risk (refer to **Table 3**). Furthermore, we converted the AIP from a continuous variable into a categorical one and reintroduced the grouped AIP into the analysis. The results from the adjusted multivariate model showed that compared to individuals in quartile 1 (Q1), the HR for those in quartiles 2 through 4 (Q2-Q4) were 0.94, 0.97, and 1.55, respectively (as shown in **Table 3**, Model II).

**Table 3:** Association between AIP and risk of diabetes in different models.

Exposure	Crude model (HR,95%CI) P	Model I(HR,95%CI) P	Model II(HR,95%CI) P	Model III(HR,95%CI) P
AIP (continuous)	6.93 (5.88, 8.16) <0.001	4.96 (4.07, 6.04) <0.001	2.07 (1.63, 2.63) <0.001	1.91 (1.47, 2.48) <0.001
AIP (Quartile)				
Q1	Ref	Ref	Ref	Ref
Q2	1.14 (0.91, 1.43) 0.254	0.98 (0.78, 1.23) 0.841	0.94 (0.75, 1.18) 0.618	0.93 (0.74, 1.17) 0.546
Q3	1.67 (1.35, 2.05) <0.001	1.24 (1.00, 1.53) 0.048	0.97 (0.78, 1.20) 0.797	0.93 (0.75, 1.15) 0.516
Q4	3.86 (3.21, 4.64) <0.001	2.47 (2.05, 2.99) <0.001	1.55 (1.27, 1.89) <0.001	1.47 (1.21, 1.80) <0.001
P for trend	<0.001	<0.001	<0.001	<0.001

Crude model: We did not adjust other covariates.

Model I: We adjusted age, gender.

Model II: We adjusted for gender, age, SBP, DBP, BMI, family history of diabetes, drinking status, smoking status, LDL-c, ALT, Scr, BUN and FPG.

Model III: We adjusted for gender, age (smooth), SBP (smooth), DBP (smooth), BMI (smooth), family history of diabetes, drinking status, smoking status, LDL-c (smooth), ALT (smooth), Scr (smooth), BUN (smooth) and FPG (smooth).

HR, Hazard ratios; CI, confidence, Ref, reference.

### Sensitivity analysis

To validate the robustness of our conclusions, we conducted a variety of sensitivity analyses. Initially, we utilized Model III of the GAM, which incorporated extra smoothing terms for various variables and resulted in a HR of 1.91 (1.47-2.48, *P*x<0.001) (refer to **Table 3**, Model III). Subsequently, we excluded individuals with systolic blood pressure (SBP) ≥140 mmHg. Adjusting for confounding variables indicated a persistent positive association between the AIP and diabetes risk (HR = 2.15, 95% CI: 1.62-2.84, *P*<0.001). In another sensitivity analysis, we removed participants with diastolic blood pressure (DBP) ≥90 mmHg. Even after adjusting for confounding factors, results continued to demonstrate a strong positive association between the AIP and diabetes risk (HR=2.02, 95% CI: 1.56-2.62, *P* < 0.001). An additional analysis focused exclusively on participants under 60 years of age yielded an HR of 2.05 (95% CI: 1.47-2.84, *P*<0.001). Our thorough sensitivity analyses reinforce the reliability of our findings (see **Table 4**). Through an in-depth analysis of the original data, we reached the same conclusion (**Supplementary Table S4**). All sensitivity analyses conducted indicated that our findings are robust. Additionally, we calculated the E-value to assess the potential impact of unmeasured confounding factors on the study results. The results showed that unknown or unmeasured variables appear to have limited influence on the relationship between AIP and diabetes risk, as the calculated E-value was 3.60, significantly higher than the relative risk value of 2.51 associated with unmeasured confounders related to AIP.

**Table 4:** Association between AIP and the risk of diabetes in different sensitivity analyses.

Exposure	Model I (HR,95%CI) P	Model II (HR,95%CI) P	Model III (HR,95%CI) P
AIP (Continuous)	2.15 (1.62, 2.84) <0.001	2.02 (1.56, 2.62) <0.001	2.05 (1.47, 2.84) <0.001
AIP (Quartile)			
Q1	Ref	Ref	Ref
Q2	0.96 (0.74, 1.24) 0.743	0.91 (0.72, 1.15) 0.4342	0.74 (0.54, 1.02) 0.065
Q3	0.87 (0.68, 1.12) 0.284	0.87 (0.69, 1.09) 0.2263	0.87 (0.65, 1.17) 0.350
Q4	1.59 (1.27, 2.00) <0.001	1.46 (1.18, 1.79) <0.001	1.53 (1.16, 2.01) 0.002
P for trend	<0.001	<0.001	<0.001

Model I was sensitivity analysis in participants with SBP<140 mmHg. We adjusted for gender, age, SBP, DBP, BMI, family history of diabetes, drinking status, smoking status, LDL-c, ALT, Scr, BUN and FPG.

Model II was sensitivity analysis in participants with DBP<90 mmHg. We adjusted for gender, age, SBP, DBP, BMI, family history of diabetes, drinking status, smoking status, LDL-c, ALT, Scr, BUN and FPG.

Model III was sensitivity analysis in participants with aged < 60 years. We adjusted for gender, age, SBP, DBP, BMI, family history of diabetes, drinking status, smoking status, LDL-c, ALT, Scr, BUN and FPG. HR, hazard ratios; CI, confidence, Ref: reference.

### Cox proportional hazards regression model with cubic spline functions to account for nonlinearity

**Figure 3:**
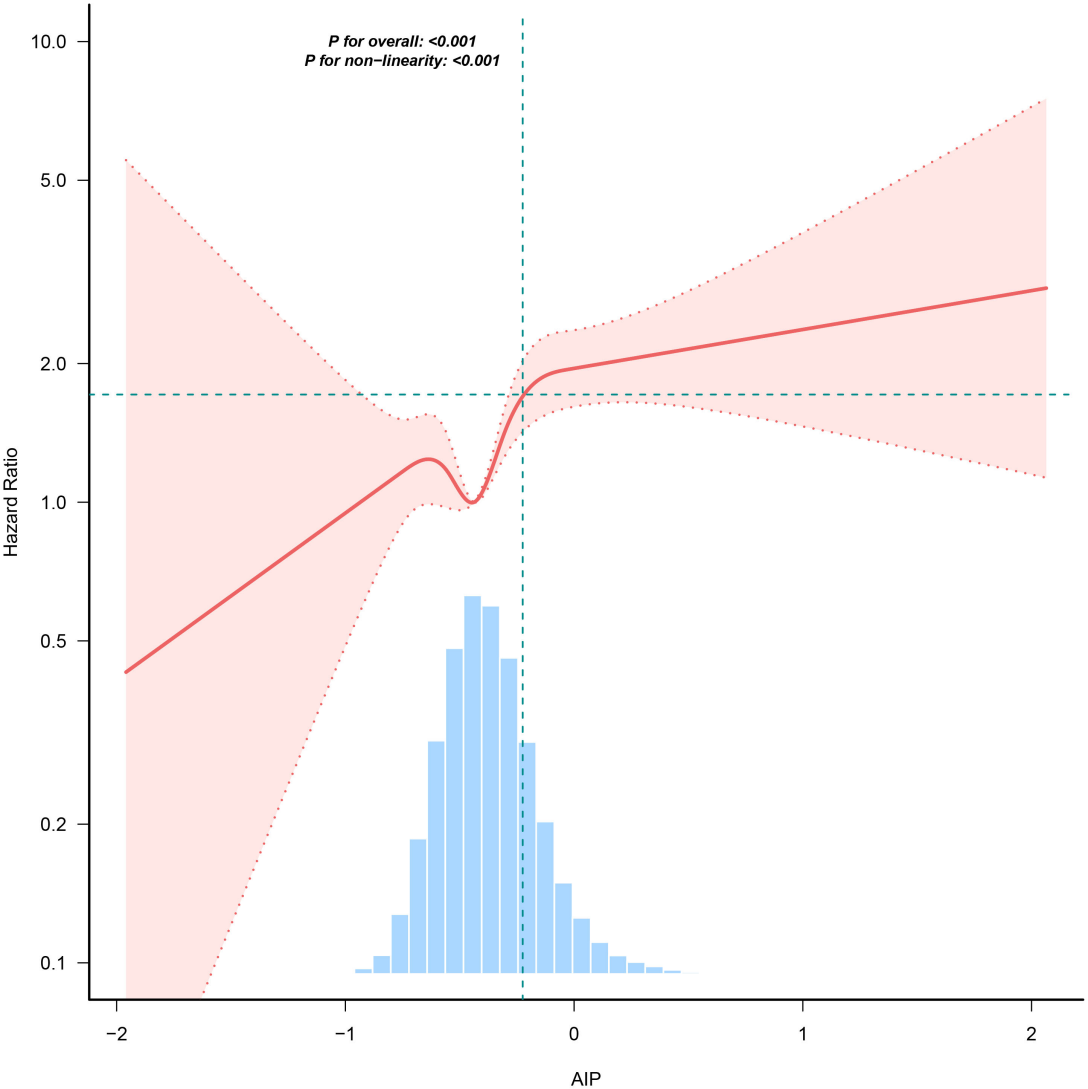
The nonlinear association between AIP and incident diabetes. A nonlinear association between them was detected after adjusting for gender, age, SBP, DBP, BMI, family history of diabetes, drinking status, smoking status, LDL-c, ALT, Scr, BUN and FPG.

In our research, we identified a connection between the AIP and diabetes risk, as illustrated in **Figure 3** and **Table 5**. We initially implemented a Cox proportional hazards regression model, utilizing cubic splines to examine the association of AIP with diabetes risk. The results indicated a non-linear association between AIP and the likelihood of developing diabetes. To further explore this association, we employed a two-piecewise Cox proportional hazards regression model. In this analysis, participants with an AIP < -0.02 demonstrated a significantly heightened risk of developing diabetes (HR 2.75, 95% CI 1.97-3.85; P <0.001), while no meaningful association was observed for those with an AIP ≥ -0.02 (HR 1.12, 95% CI 0.62-2.02; P = 0.709). The p-value from the log-likelihood ratio test, which evaluated the overall fit of the two-piecewise model, was found to be 0.016, suggesting a non-linear association between AIP and diabetes risk.

**Table 5:** The result of the two-piecewise Cox proportional hazards regression model.

Incident diabetes	HR (95%CI)	P-value
Fitting model by standard Cox proportional hazards regression	2.07 (1.63, 2.63)	<0.001
Fitting model by two-piecewise Cox proportional hazards regression		
Inflection points of AIP	-0.02	
<-0.02	2.75 (1.97, 3.85)	<0.001
≥-0.02	1.12 (0.62, 2.02)	0.709
P for log likelihood ratio test		0.016

HR, Hazard ratios; CI, confidence. We adjusted gender, age, SBP, DBP, BMI, family history of diabetes, drinking status, smoking status, LDL-c, ALT, Scr, BUN and FPG.

### Subgroup analysis

**Figure 4:**
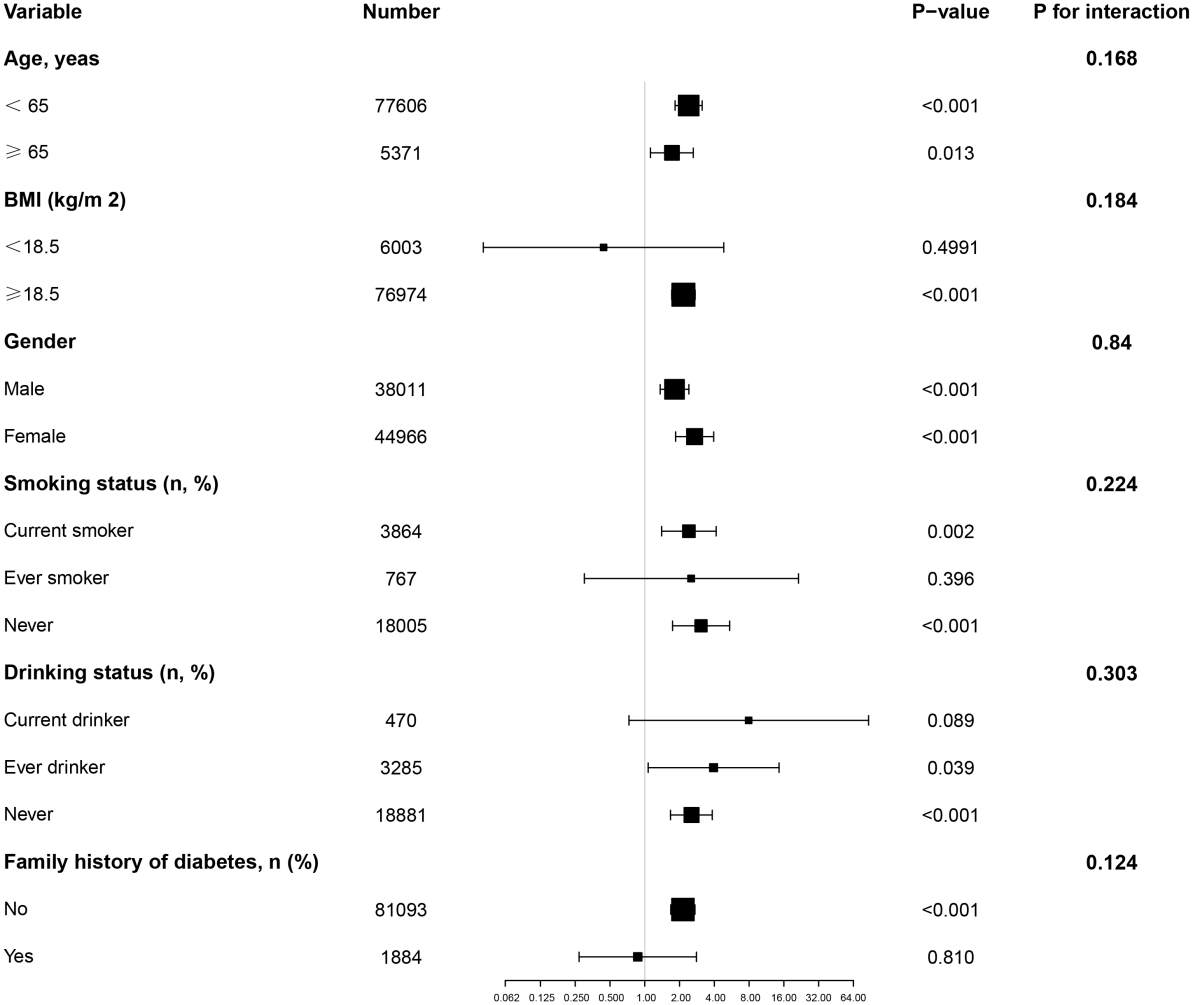
Effect size of AIP on diabetes in prespecified and exploratory subgroups. Note: Above model adjusted for gender, age, SBP, DBP, BMI, family history of diabetes, drinking status, smoking status, LDL-c, ALT, Scr, BUN and FPG. In each case, the model is not adjusted for the stratification variable.

In all predefined or exploratory subgroup analyses (**Figure 4**), gender, BMI, age, smoking status, drinking, and family history of diabetes did not alter the relationship between AIP and diabetes risk. In other words, there were no significant interactions between these factors and AIP (interaction P > 0.05).

## Discussion

This is a large retrospective cohort study aimed at exploring the association between the AIP and future diabetes risk in non-obese populations in China. Our study found that baseline AIP was positively correlated with diabetes risk, which remained significant after adjusting for other covariates. Additionally, there was a non-linear relationship between AIP and diabetes risk, with the study identifying a threshold value of -0.02 for AIP. Specifically, participants with an AIP below -0.02 exhibited a significantly increased risk of developing diabetes, whereas the risk of developing diabetes did not significantly increase with AIP among those with values above -0.02.

According to a national survey, the prevalence of diabetes in China was 12.8% in 2017, and the incidence rate is increasing (27). However, the incidence of diabetes observed in this study was lower than that reported in the general population. This discrepancy may be attributed to the predominantly nature of the study participants without obesity, who were at lower risk for diabetes due to the absence of conditions such as hypertension and coronary heart disease. Additionally, the median follow-up period of 3.10 years in this study was relatively short, during which the incidence of diabetes remained modest. It is noteworthy that despite a predominantly younger cohort, the prevalence of diabetes was still 1.25%, consistent with findings by Cai et al. (28). Therefore, identifying risk factors predisposing individuals to diabetes remains crucial. Screening for these risk factors and early intervention are essential for preventing disease progression and adverse outcomes.

Previous studies consistently indicate that dyslipidemia characterized by elevated triglyceride levels and decreased high-density lipoprotein cholesterol (HDL-C) is a significant feature of the diabetes-prone environment or diabetes patients (29, 30). The concept of using the TG/HDL-C ratio to assess insulin resistance was proposed early on (31–33). In recent years, numerous studies have elucidated a significant association between TG/HDL-C and diabetes events (34–36). AIP represents the logarithm of the TG/HDL-C ratio. Recent research has found a significant association between AIP and diabetes risk. Yin et al. conducted a retrospective cross-sectional study involving 9,245 U.S. adults, revealing a positive association between AIP and both insulin resistance (IR) and type 2 diabetes, showing a non-linear association (37). Another retrospective cohort study in the United States utilized data from the National Health and Nutrition Examination Survey (NHANES) from 2011 to 2018, analyzing 10,099 adults, demonstrating a significant association between increasing AIP and the prevalence of prediabetes and diabetes, with stronger associations observed in females (38). A retrospective cohort study of 585 Korean pregnant women highlighted a significant positive association between AIP and gestational diabetes mellitus (GDM). After multivariable adjustment, every 0.1-unit increase in AIP was associated with a 58% increased risk of GDM and demonstrated good predictive capability (39). Yi et al., in a retrospective cohort study of 8,760 Chinese adults aged over 45 years, found a close association between AIP and diabetes incidence (40). Interestingly, a retrospective study in Henan, China, involving 40,633 adult participants with overweight or obesity, found a significant association between AIP and the risk of type 2 diabetes (T2DM). Individuals with higher AIP levels (Q4 group) had a higher risk of T2DM compared to those with lower AIP levels (Q1 group) (OR = 5.17, 95% CI 4.69–5.69). Additionally, the association between AIP and T2DM showed a non-linear association that weakened with increasing age (41).

However, there is limited research on the association between AIP and diabetes among non-obese populations. Nonetheless, the incidence of diabetes in this group is gradually increasing. Therefore, we enrolled 82,977 Chinese non-obese participants in a long-term longitudinal cohort study. Our study found a significant positive association between AIP and the likelihood of developing diabetes. The risk of diabetes increased across quartiles of AIP, with individuals in the highest quartile (Q4) showing a notably higher risk compared to those in the lowest quartile (Q1). Furthermore, we identified a non-linear association between AIP and diabetes risk in non-obese adults. Specifically, participants with an AIP < -0.02 demonstrated a significantly increased risk of developing diabetes, while no meaningful association was observed for those with an AIP ≥ -0.02. This is similar to the results of most studies, but there are also essential differences (37–41). First, this study found that in non-obese populations, there is a saturation effect between AIP and the likelihood of developing diabetes, whereas previous studies mostly suggested a threshold effect between the two (37, 41). Second, in overweight and obese populations, the incidence of diabetes remains low when AIP is below -0.07 (41). Based on the results of this study, non-obese individuals need to maintain AIP at -0.02 or lower to reduce the incidence of diabetes. This indicates that non-obese populations need to have stricter control over AIP levels to lower the incidence of diabetes.

This study has several highlights. First, we are the first to investigate the association between AIP and diabetes risk in non-obese individuals in China. The results indicate that in this population, AIP is non-linearly related to diabetes risk and exhibits a saturation effect. Furthermore, this study includes a large sample size, which enhances the statistical power and reliability of the findings. To ensure the validity and robustness of the results, we conducted sensitivity analyses, subgroup analyses, and interaction tests. Finally, this study also calculated the E-value to assess the influence of unmeasured factors on the outcomes.

Nonetheless, it is important to recognize that this research may encounter certain limitations. First, due to its retrospective nature, the researchers were unable to manage the aspects of data gathering and documentation, which might have resulted in incomplete or erroneous data acquisition. Furthermore, retrospective research is susceptible to biases related to selection and information. Second, since the analysis relies on pre-existing published data, the quality of this original information may influence the accuracy and dependability of the findings. Third, the participants involved in the study were solely from Asia, with a concentration on China, possibly constraining the applicability of the results to other regions around the world. Therefore, additional studies should be conducted to explore the association between AIP and the risk of diabetes across various areas, including the Middle East and India. Moreover, the researchers were unable to control for numerous variables during data collection, which could encompass confounding elements or neglected factors that might impact the integrity of the analytical outcomes. But we calculated the E-value to assess sensitivity to unmeasured confounding factors and found that the influence of unknown or unmeasured variables on the association between AIP and diabetes risk appears to be minimal. Finally, given that this research is essentially observational, it establishes a hypothetical connection between AIP and diabetes risk rather than demonstrating a definitive causal association. Therefore, we strongly recommend conducting prospective longitudinal studies in the future. Such studies could help us further understand the direct causal association between the modulation of AIP levels and the reduction of diabetes risk.

## Conclusion

Our study reveals a positive correlation between baseline AIP and diabetes risk, and this relationship exhibits a non-linear saturation effect. Specifically, when the AIP value is below -0.02, participants show a significantly increased risk of developing diabetes. However, for participants with an AIP value equal to or greater than -0.02, the risk of developing diabetes does not show a significant increase. Understanding this non-linear association provides important evidence for clinicians, enabling them to effectively identify high-risk individuals and implement targeted interventions to reduce the risk of developing diabetes to some extent.

## Data Availability

The datasets presented in this study can be found in online repositories. The names of the repository/repositories and accession number(s) can be found in the article/**Supplementary Material**.
