# Supplementary material for: The atherogenic index of plasma is associated with an increased risk of diabetes in non-obese adults: a cohort study
Source: Front Endocrinol (Lausanne). 2025 Jan 20;15:1477419. doi: 10.3389/fendo.2024.1477419 (PMC11788137; doi:10.3389/fendo.2024.1477419)
Supplement: Supplementary file 1 [file DataSheet1.docx]

**The atherogenic index of plasma is associated with an increased risk of diabetes in adults without obesity：a cohort study**

**Running title:** AIP is associated with risk of diabetes

Jun Cao^1^, Zhaohai Su^1^, Jiangyong Yang^1^, Bilong Zhang^1^, Rengui Jiang^1^, Weiling Lu^1^, Zhenhua Huang^2*^, Zheng Xie^3*^

**Affiliation**

^1^Department of Cardiology, Guangdong Provincial People's Hospital Ganzhou Hospital, Ganzhou Municipal Hospital（Gannan Medical University Affiliated Municipal Hospital）, 49 Dagong Road, 341000, Ganzhou, China.

^2^ Department of Emergency Medicine, the First Affiliated Hospital of Shenzhen University, Shenzhen Second People’s Hospital, Shenzhen, 518035, China. email: huangzhh12306@163.com.

^3^ Department of General Practice, Guangdong Provincial People's Hospital Ganzhou Hospital, Ganzhou Municipal Hospital（Gannan Medical University Affiliated Municipal Hospital）, 49 Dagong Road, 341000, Ganzhou, China.

**^*^Corresponding author information**

**Zhenhua Huang, PD**

Department of Emergency Medicine, the First Affiliated Hospital of Shenzhen University, Shenzhen Second People’s Hospital, Shenzhen, 518035, China. E-mail addresses: huangzhh12306@163.com.

**Zheng Xie, PD**

Department of Cardiology, Ganzhou Hospital of Guangdong Provincial People's Hospital, Ganzhou Municipal Hospital（Gannan Medical University Affiliated Municipal Hospital）, Ganzhou, China, 49 Dagong Road,341000 Ganzhou, E-mail addresses: [shottome1@163.com](mailto:shottome1@163.com).

**Table S1** Collinearity screening

|  | Step 1 | Step 2 |
| --- | --- | --- |
| BMI (kg/m^2^) | 1.3 | 1.3 |
| Age(years) | 1.4 | 1.3 |
| SBP (mmHg) | 2.1 | 2.1 |
| DBP (mmHg) | 1.9 | 1.9 |
| TC (mmol/L) | 8.1 | NA |
| TG (mmol/L) | 5.1 | 4.8 |
| HDL-c(mmol/L) | 1.7 | 1.2 |
| LDL-c(mmol/L) | 7.2 | 1.7 |
| ALT(U/L) | 1.1 | 1.1 |
| FPG (mmol/L) | 1.1 | 1.1 |
| BUN (mmol/L) | 1.2 | 1.2 |
| Scr (μmol/L) | 2.2 | 2.2 |
| Smoking status | 3.2 | 3.2 |
| Drinking status | 3.2 | 3.2 |
| Family history of diabetes | 1 | 1 |
| Gender | 2.4 | 2.4 |

Variables excluded from collinearity screening: TC

Abbreviations: DBP, diastolic blood pressure; BMI, body mass index; TC, total cholesterol, SBP, systolic blood pressure; TG triglyceride, BMI, body mass index; AST aspartate aminotransferase; LDL-c, low-density lipid cholesterol; ALT, alanine aminotransferase; BUN, blood urea nitrogen; HDL-c, high-density lipoprotein cholesterol; Scr, serum creatinine.

**Table S2** The baseline characteristics of participants between DM and Non-DM.

| Group | Non-DM | DM | *P*-value |
| --- | --- | --- | --- |
| participants | 81,936 | 1,041 |  |
| Age (years) | 42.84 ± 12.70 | 58.40 ± 12.76 | <0.001 |
| BMI (kg/m^2^) | 21.68 ± 2.04 | 22.77 ± 1.68 | <0.001 |
| SBP (mmHg) | 116.27 ± 15.67 | 129.43 ± 19.66 | <0.001 |
| DBP (mmHg) | 72.40 ± 10.20 | 78.19 ± 11.58 | <0.001 |
| FBG (mmol/L) | 4.87 ± 0.57 | 5.90 ± 0.74 | <0.001 |
| TC (mmol/L) | 4.71 ± 0.88 | 5.08 ± 0.98 | <0.001 |
| TG (mmol/L) | 1.17 ± 0.82 | 1.81 ± 1.29 | <0.001 |
| HDL-c (mmol/L) | 1.42 ± 0.31 | 1.36 ± 0.41 | <0.001 |
| LDL-c (mmol/L) | 2.72 ± 0.67 | 2.91 ± 0.72 | <0.001 |
| ALT (U/L) | 19.93 ± 18.40 | 25.53 ± 18.00 | <0.001 |
| Scr (μmol/L) | 68.24 ± 15.37 | 70.98 ± 16.63 | <0.001 |
| BUN (mmol/L) | 4.60 ± 1.17 | 5.05 ± 1.32 | <0.001 |
| Gender (n, %) |  |  | <0.001 |
| Male | 37369 (45.61%) | 642 (61.67%) |  |
| Female | 44567 (54.39%) | 399 (38.33%) |  |
| Smoking status (n, %) |  |  | <0.001 |
| Current smoker | 3774 (4.61%) | 90 (8.65%) |  |
| Ever smoker | 754 (0.92%) | 13 (1.25%) |  |
| Never | 17820 (21.75%) | 185 (17.77%) |  |
| Unknown | 59588 (72.73%) | 753 (72.33%) |  |
| Drinking status (n, %) |  |  | 0.119 |
| Current drinker | 465 (0.57%) | 5 (0.48%) |  |
| Ever drinker | 3257 (3.98%) | 28 (2.69%) |  |
| Never | 18626 (22.73%) | 255 (24.50%) |  |
| Unknown | 59588 (72.73%) | 753 (72.33%) |  |
| Family history of diabetes, n (%) | 1837 (2.24%) | 47 (4.51%) | <0.001 |
| Follow-up (year) | 3.10 ± 0.95 | 3.35 ± 0.97 | <0.001 |

Continuous variables were summarized as mean (SD) or medians (quartile interval); categorical variables were displayed as percentage (%). Abbreviations: BMI, body mass index; SBP, systolic blood pressure; DBP; diastolic blood pressure; TG triglyceride; ALT, alanine aminotransferase; BUN, blood urea nitrogen; Scr, serum creatinine; FBG, fasting plasma glucose; TC, total cholesterol; HDL-c, high-density lipoprotein cholesterol; LDL-c, low-density lipoprotein cholesterol.

**Table S3** Risk of diabetes analyzed by univariate Cox proportional hazards regression.

| Variable | Characteristics | HR (95% CI) | *P*-value | |
| --- | --- | --- | --- | --- |
| Age (years) | 43.04 ± 12.82 | 1.08 (1.07, 1.08) | <0.001 |  |
| Gender (n, %) |  |  |  |  |
| Male | 38011 (45.81%) | 1.0 |  |  |
| Female | 44966 (54.19%) | 0.53 (0.46, 0.60) | <0.001 |  |
| BMI (kg/m^2^) | 21.69 ± 2.04 | 1.35 (1.30, 1.40) | <0.001 |  |
| SBP (mmHg) | 116.44 ± 15.79 | 1.04 (1.04, 1.04) | <0.001 |  |
| DBP (mmHg) | 72.48 ± 10.23 | 1.05 (1.04, 1.05) | <0.001 |  |
| FPG (mmol/L) | 4.88 ± 0.58 | 12.41 (11.38, 13.54) | <0.001 |  |
| TC (mmol/L) | 4.71 ± 0.88 | 1.48 (1.40, 1.57) | <0.001 |  |
| TG (mmol/L) | 1.18 ± 0.83 | 1.29 (1.27, 1.32) | <0.001 |  |
| AIP | -0.42 ± 0.23 | 6.93 (5.88, 8.16) | <0.001 |  |
| ALT (U/L) | 20.00 ± 18.41 | 1.00 (1.00, 1.00) | <0.001 |  |
| LDL-c (mmol/L) | 2.72 ± 0.67 | 1.52 (1.40, 1.64) | <0.001 |  |
| HDL-c (mmol/L) | 1.42 ± 0.31 | 0.72 (0.59, 0.88) | 0.001 |  |
| Scr (μmol/L) | 68.28 ± 15.39 | 1.01 (1.01, 1.01) | <0.001 |  |
| BUN (mmol/L) | 4.61 ± 1.17 | 1.32 (1.26, 1.37) | <0.001 |  |
| **Smoking status (n, %)** |  |  |  |  |
| Current smoker | 3864 (4.66%) | 1.0 |  |  |
| Ever smoker | 767 (0.92%) | 0.70 (0.39, 1.26) | 0.235 |  |
| Never | 18005 (21.70%) | 0.45 (0.35, 0.58) | <0.001 |  |
| Unknown | 60341 (72.72%) | 0.57 (0.46, 0.71) | <0.001 |  |
| **Drinking status (n, %)** |  |  |  |  |
| Current drinker | 470 (0.57%) | 1.0 |  |  |
| Ever drinker | 3285 (3.96%) | 0.62 (0.24, 1.61) | 0.330 |  |
| Never | 18881 (22.75%) | 1.05 (0.43, 2.55) | 0.911 |  |
| Unknown | 60341 (72.72%) | 1.00 (0.42, 2.42) | 0.993 |  |
| **Family history of diabetes, n (%)** |  |  |  |  |
| No | 81093 (97.73%) | 1.0 |  |  |
| Yes | 1884 (2.27%) | 1.71 (1.27, 2.29) | <0.001 |  |

HR, Hazard ratios; CI, confidence, Ref, reference.

**Table S4** Relationship between AIP and risk of diabetes in different models in the raw data

| Exposure | Crude model (HR,95%CI) P | Model I(HR,95%CI) P | Model II(HR,95%CI) P |
| --- | --- | --- | --- |
| AIP (continuous) | 6.9 (5.9, 8.2) <0.001 | 5.0 (4.1, 6.0) <0.001 | 2.0 (1.6, 2.6) <0.001 |
| AIP (Quartile) |  |  |  |
| Q1 | Ref | Ref | Ref |
| Q2 | 1.1 (0.9, 1.4) 0.254 | 1.0 (0.8, 1.2) 0.841 | 1.0 (0.8, 1.2) 0.726 |
| Q3 | 1.7 (1.4, 2.1) <0.001 | 1.2 (1.0, 1.5) 0.048 | 0.9 (0.7, 1.1) 0.445 |
| Q4 | 3.9 (3.2, 4.6) <0.001 | 2.5 (2.1, 3.0) <0.001 | 1.5 (1.2, 1.9) <0.001 |
| P for trend | <0.001 | <0.001 | <0.001 |

Crude model: We did not adjust other covariates.

Model I: We adjusted age, gender.

Model II: We adjusted for gender, age, SBP, DBP, BMI, family history of diabetes, drinking status, smoking status, TC, LDL-C, ALT, Scr, BUN and FPG


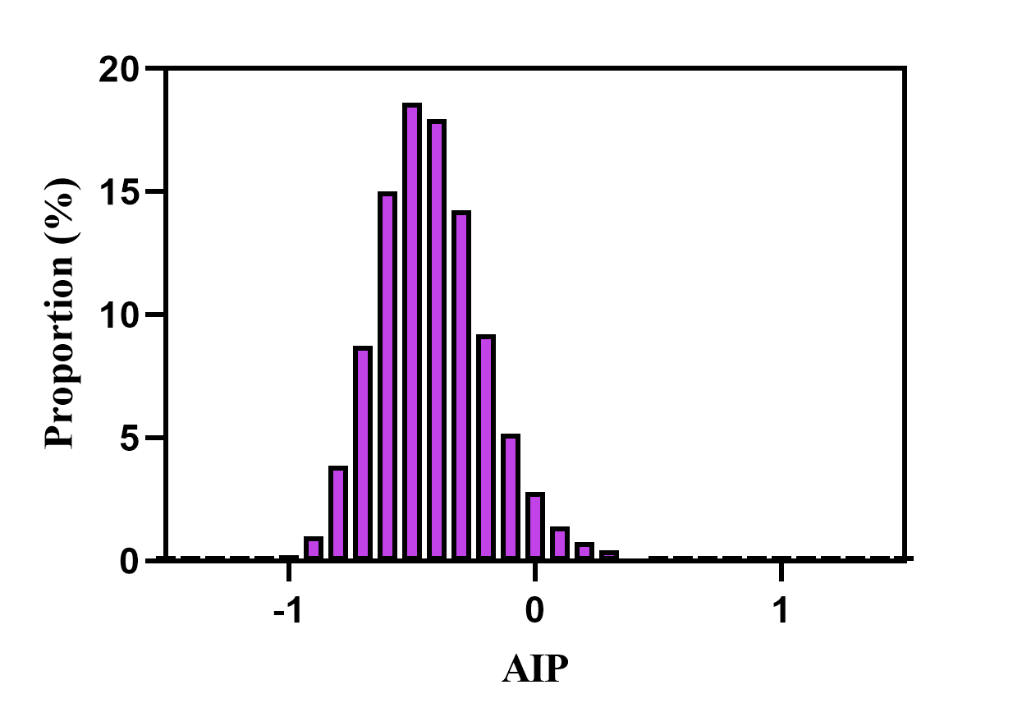


**Figure S1** Distribution of AIP. It showed that AIP for merge individuals presented a normal distribution ranging from -1.0 to 1.47, with a mean level of -0.163.


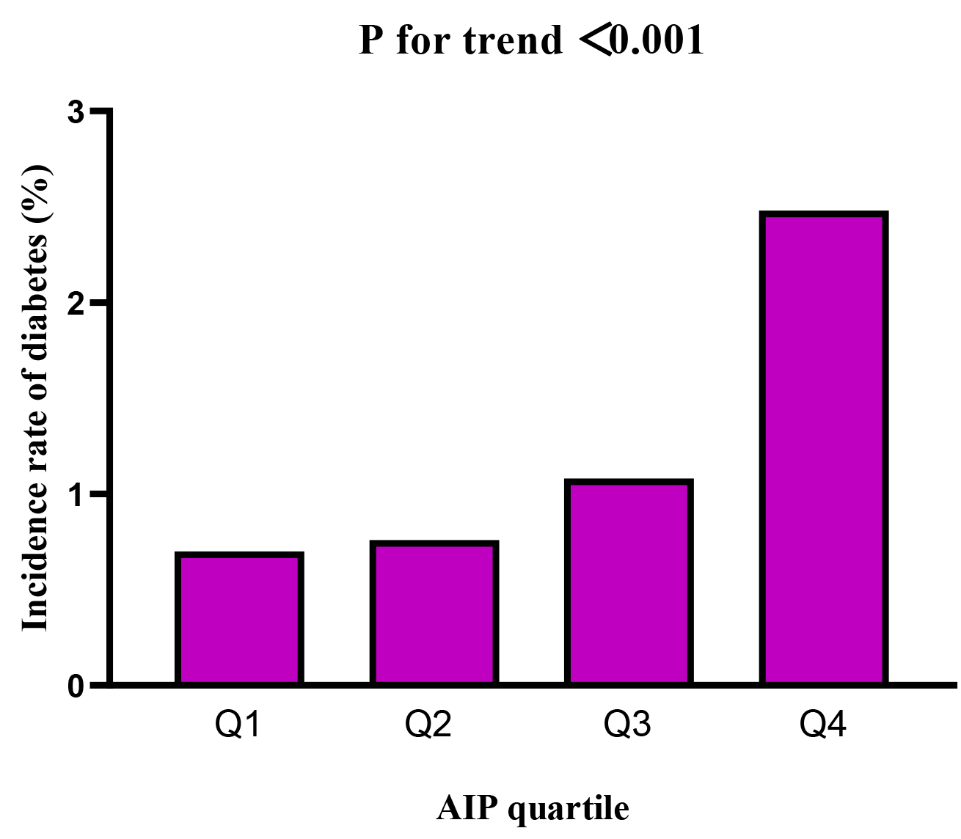


# Figure S2 The incidence rate of diabetes according to the quartiles of AIP. Participants with the highest AIP (Q4) had higher rate of diabetes than those with the lowest AIP (Q1) *P* < 0.001 for trend)
